# Win percentage: a novel measure for assessing the suitability of machine classifiers for biological problems

**DOI:** 10.1186/1471-2105-13-S3-S7

**Published:** 2012-03-21

**Authors:** R Mitchell Parry, John H Phan, May D Wang

**Affiliations:** 1 The Wallace H Coulter Department of Biomedical Engineering, Georgia Institute of Technology and Emory University, Atlanta, GA 30332, USA; 2 Department of Electrical and Computer Engineering, Georgia Institute of Technology, Atlanta, GA 30332, USA; 3 Winship Cancer Institute, Emory University, Atlanta, GA 30322, USA; 4 Parker H Petit Institute of Bioengineering and Biosciences, Georgia Institute of Technology, Atlanta, GA 30332, USA

## Abstract

**Background:**

Selecting an appropriate classifier for a particular biological application poses a difficult problem for researchers and practitioners alike. In particular, choosing a classifier depends heavily on the features selected. For high-throughput biomedical datasets, feature selection is often a preprocessing step that gives an unfair advantage to the classifiers built with the same modeling assumptions. In this paper, we seek classifiers that are suitable to a particular problem independent of feature selection. We propose a novel measure, called "win percentage", for assessing the suitability of machine classifiers to a particular problem. We define win percentage as the probability a classifier will perform better than its peers on a finite random sample of feature sets, giving each classifier equal opportunity to find suitable features.

**Results:**

First, we illustrate the difficulty in evaluating classifiers after feature selection. We show that several classifiers can each perform statistically significantly better than their peers given the right feature set among the top 0.001% of all feature sets. We illustrate the utility of win percentage using synthetic data, and evaluate six classifiers in analyzing eight microarray datasets representing three diseases: breast cancer, multiple myeloma, and neuroblastoma. After initially using all Gaussian gene-pairs, we show that precise estimates of win percentage (within 1%) can be achieved using a smaller random sample of all feature pairs. We show that for these data no single classifier can be considered the best without knowing the feature set. Instead, win percentage captures the non-zero probability that each classifier will outperform its peers based on an empirical estimate of performance.

**Conclusions:**

Fundamentally, we illustrate that the selection of the most suitable classifier (*i.e*., one that is more likely to perform better than its peers) not only depends on the dataset and application but also on the thoroughness of feature selection. In particular, win percentage provides a single measurement that could assist users in eliminating or selecting classifiers for their particular application.

## Background

Machine classifiers and feature selection algorithms have been proposed for clinical diagnosis and prediction based on favorable comparisons to competing methods [1,2]. For high-throughput biomedical data, feature selection is a necessary preprocessing or embedded step that can bias the comparison of classifiers. In an effort to compare classifiers fairly, we introduce the idea of classifier "suitability" to a particular application. Every classifier is more or less suited to model particular feature relationships. For example, linear classifiers anticipate modeling features exhibiting a mean shift between classes, whereas nonlinear classifiers can model more complex corner shapes or quadratic curves [3]. Classifier suitability depends on two key aspects: (1) how frequently the feature relationships it models discriminate between classes in the data and (2) how thoroughly we explore the feature space to find those relationships. We propose "win percentage" as the probability that a classifier will perform better than its peers on a finite random sample of feature sets.

As an analytical tool to aid in our estimation of win percentage, we design a Monte Carlo wrapper (MCW) algorithm for feature selection that gives each classifier equal opportunity to find informative feature sets. MCW succeeds when its best-performing feature set is among a top-performing fraction of all possible feature sets. This fraction, combined with a tolerated failure rate, defines the number of random samples that MCW must explore. We show that the most suitable classifier for an application depends on how thoroughly we explore the feature space, and apply win percentage in the analysis of eight biomedical gene expression classification problems^a ^[4].

Determining the most suited classifier to a particular problem has applications in many domains but we are most interested in the translation of machine learning algorithms for clinical diagnosis and prediction. Ideally, an exhaustive search of all feature sets could identify the optimal feature set for each classifier. However, for high-throughput biomedical data many thousands of features make this infeasible and necessitate the use of feature selection methods. A multitude of computationally efficient, yet suboptimal, feature selection methods have been proposed [5,6] but these have made comparing the resulting learning machines more difficult and potentially exclude otherwise suitable feature sets. Often, the same feature selection method precedes the comparison of all classifiers using cross-validation. However, the performance of a classifier depends on the feature selection method that precedes it. One way to deal with this inherent dependency is to consider a combinatorial approach of feature selection methods and classifiers, selecting combinations of both that perform well on cross-validation [7,8]. Another way is to attempt to find a feature selection method that performs well for a variety of typical datasets [3]. We simplify both approaches by considering a single unbiased feature selection method that gives every classifier equal chance to perform well. Instead of finding a classifier that performs well for a given feature selection method, we attempt to identify classifiers that fit the problem.

Feature selection methods can be categorized into filter- and wrapper-based approaches. Filter-based methods rank genes based on some measure of utility such as the difference between class means (e.g., *t*-test p-value or fold-change). This emphasis on class means favors linear classifiers that consider the mean as the single distinguishing characteristic among classes (*e.g.*, nearest centroid). However, nonlinear classifiers have been shown to perform well for a variety of problems [9] and deserve equal treatment when it comes to feature selection. Wrapper-based feature selection attempts to find feature sets that perform well for a particular classifier using that classifier as a black box [10]. Several heuristic wrapper-based feature selection methods are commonly used for nonlinear classifiers, such as sequential forward selection or backward elimination [6]. However, these suffer from a nesting structure that causes all explored feature sets to contain highly overlapping feature membership. One way to give each classifier an equal chance of finding a suitable feature set is to conduct a *randomized *search of the feature space using a wrapper-based approach. The classification performance of each candidate classifier determines the quality of a feature set.

Randomized algorithms come in two basic varieties: those that provide the correct answer for a given input every time (Las Vegas), and those that may give different answers to the same problem on multiple runs (Monte Carlo) [11]. Las Vegas algorithms have been proposed for feature selection [5,12] but have fallen out of favor perhaps due to the relative success of faster heuristic methods. Monte Carlo feature selection has been used to select features that commonly appear in different cross-validation runs [13]. Stochastic algorithms such as simulated annealing and genetic algorithms offer a compromise in that previous results guide the search but maintain randomness to avoid local optima [14].

Regardless of feature selection method, the utility of a particular classifier depends not only on its performance on a carefully selected feature set but also on the difficulty in discovering that feature set. That is, depending on computational resources and time, the most suitable classifier may change. By randomly sampling feature sets, we remove classifier bias and separate the comparison of classifiers from feature selection. Although this approach requires significantly more computational resources than heuristic methods, it provides a foundation for a fair comparison between classifiers.

## Results

First, we motivate our study by illustrating that each classifier appears to perform better than its peers for each dataset given the right feature set. Therefore, the difficulty in finding the right feature set must play a role in determining the suitability of a classifier. Second, we show that win percentage accomplishes this goal in a simple example, and demonstrate the correspondence between the continuous version of our win percentage and the discrete version. After demonstrating the utility of our approach using synthetic datasets from known distributions, we apply it to analyze datasets from the FDA MAQC-II Project [15].

### Demonstrating the utility of each classifier for each dataset

**Figure 1:**
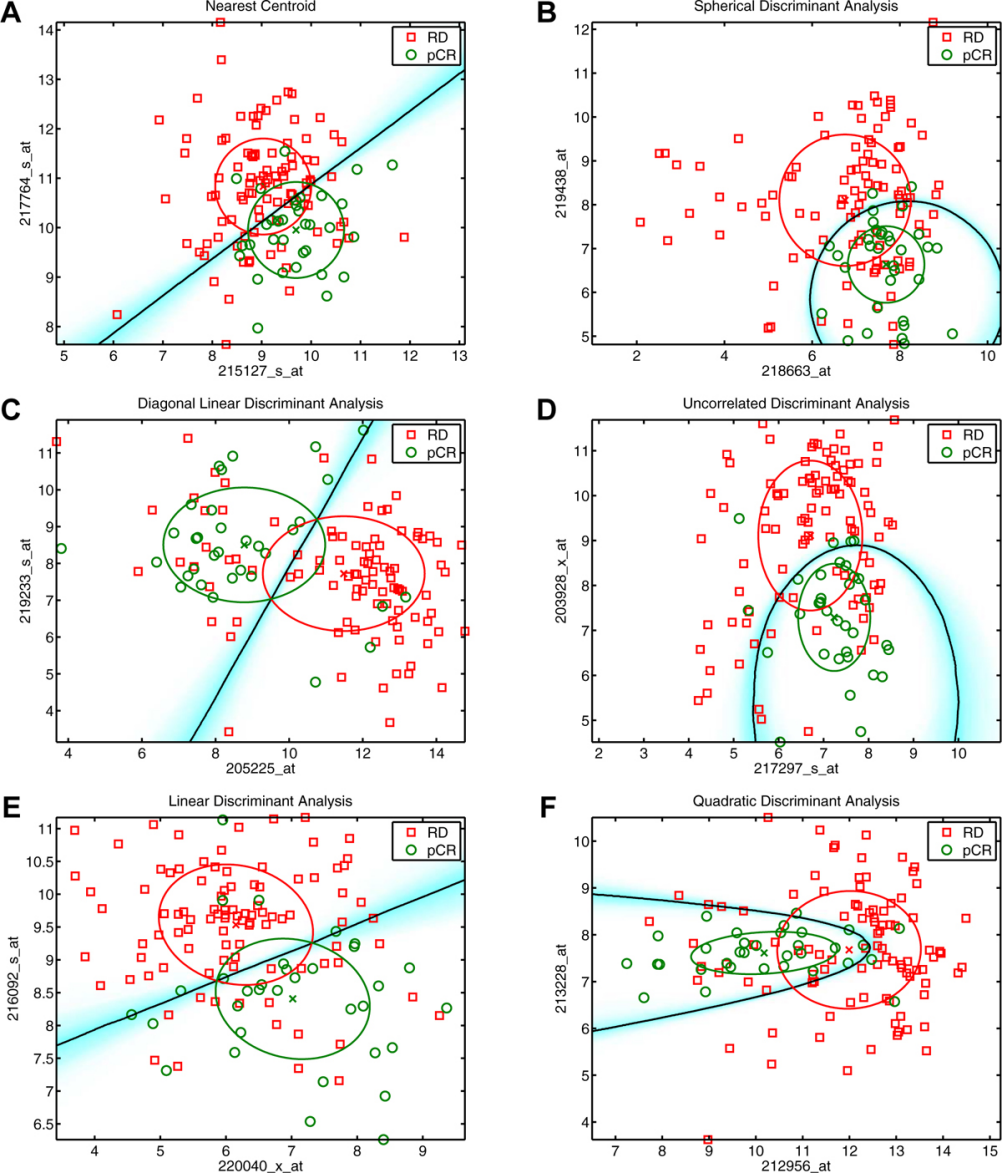
**Classifier discrimination plots for breast cancer, pathological complete response.** Panels A, B, C, D, E, and F correspond to the classifiers, NC, SDA, DLDA, UDA, LDA, and QDA, respectively. RD indicates residual invasive tumor, and pCR indicates pathological complete response. The black line indicates the average decision boundary across 100 folds of cross-validation. The cyan shading indicates the uncertainty in the labeling. White areas are labeled the same for every fold, whereas dark cyan represents the most uncertainty. The ellipses correspond to one standard deviation away from the mean for each class indicated with an 'x'.

Figure 1 provides scatter plots for selected feature sets that exemplify the utility of each classifier. The black line or curve in each panel represents the average decision boundary across 20 iterations of five-fold cross-validation. The white background indicates regions of the feature space that receive a unanimous label for all 20 iterations of cross-validation. The cyan shading represents the uncertainty of sample labeling reaching a peak at the decision boundary. The red and green ellipses mark a distance one standard deviation away from the mean (marked with an 'x') of each class. Each panel corresponds to one row in Table 1. For example, panel A corresponds to the first row showing the two gene names from the plot and that nearest centroid achieved a performance of 0.812. The remaining classifiers performed significantly worse and therefore do not carry an asterisk. In fact, all six classifiers (panel A - F of Figure 1) showed significantly better performance than their peers given the right feature set. Nearly all classifiers showed significantly better performance for all datasets given the right high performing (top 100) feature set. Figures S1-6 and Tables S1-6 in Additional File 1 provide scatter plots and performance results for all cancer datasets.

**Table 1:** Estimated classifier performance for breast cancer, pathological complete response

Gene 1	Gene 2	NC	DLDA	LDA	SDA	UDA	QDA
217764_s_at	215127_s_at	0.812*	0.774	0.771	0.752	0.743	0.748
219233_s_at	205225_at	0.787	0.818*	0.770	0.781	0.799	0.775
216092_s_at	220040_x_at	0.785	0.787	0.796*	0.782	0.782	0.776
219438_at	218663_at	0.761	0.763	0.762	0.806*	0.777	0.771
203928_x_at	217297_s_at	0.739	0.744	0.759	0.793	0.810*	0.802
213228_at	212956_at	0.764	0.761	0.761	0.765	0.799	0.809*

* Top performing classifier and any classifiers that do not have a significantly lower mean using a paired *t*-test with *α *= 0.05.

### An illustrative example of win percentage

To illustrate our approach, we simulate experimental data starting with Gaussian conditional distributions *p*(*x*|*c*) for three candidate classifiers with the following parameters:

(1) $$\begin{array}{cc} p\left(x|C=c_{1}\right) & =N\left(0.50,0.20\right),\\ p\left(x|C=c_{2}\right) & =N\left(0.70,0.07\right),\\ p\left(x|C=c_{3}\right) & =N\left(0.75,0.02\right), \end{array}\begin{array}{cc} \;p\left(C=c_{1}\right) & =1/3\\ \;p\left(C=c_{2}\right) & =1/3.\\ \;p\left(C=c_{2}\right) & =1/3 \end{array}$$

**Figure 2:**
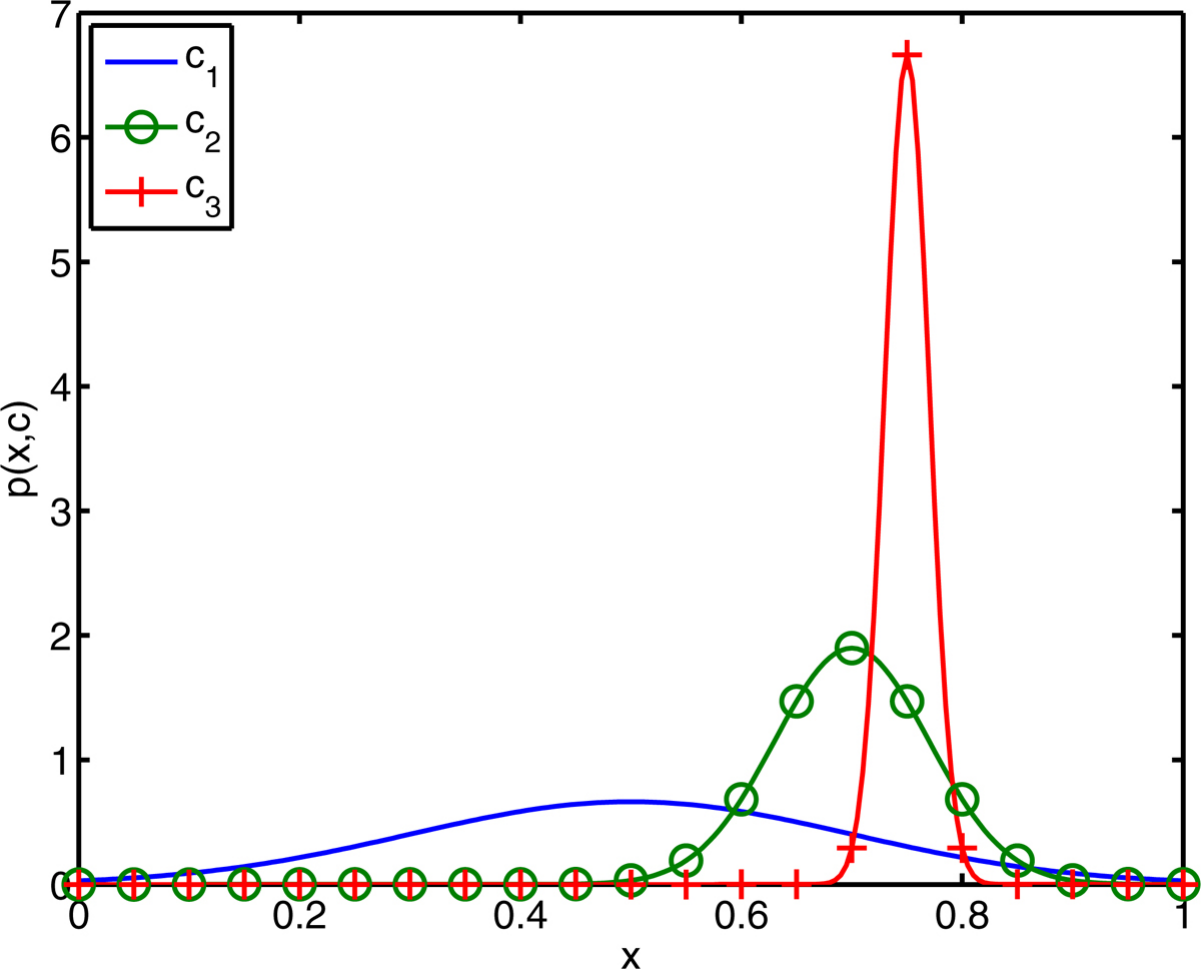
**Distribution of winning classifier performance.** The joint probability density functions for the classifiers in the illustrative example defined in Equation 1.

**Figure 3:**
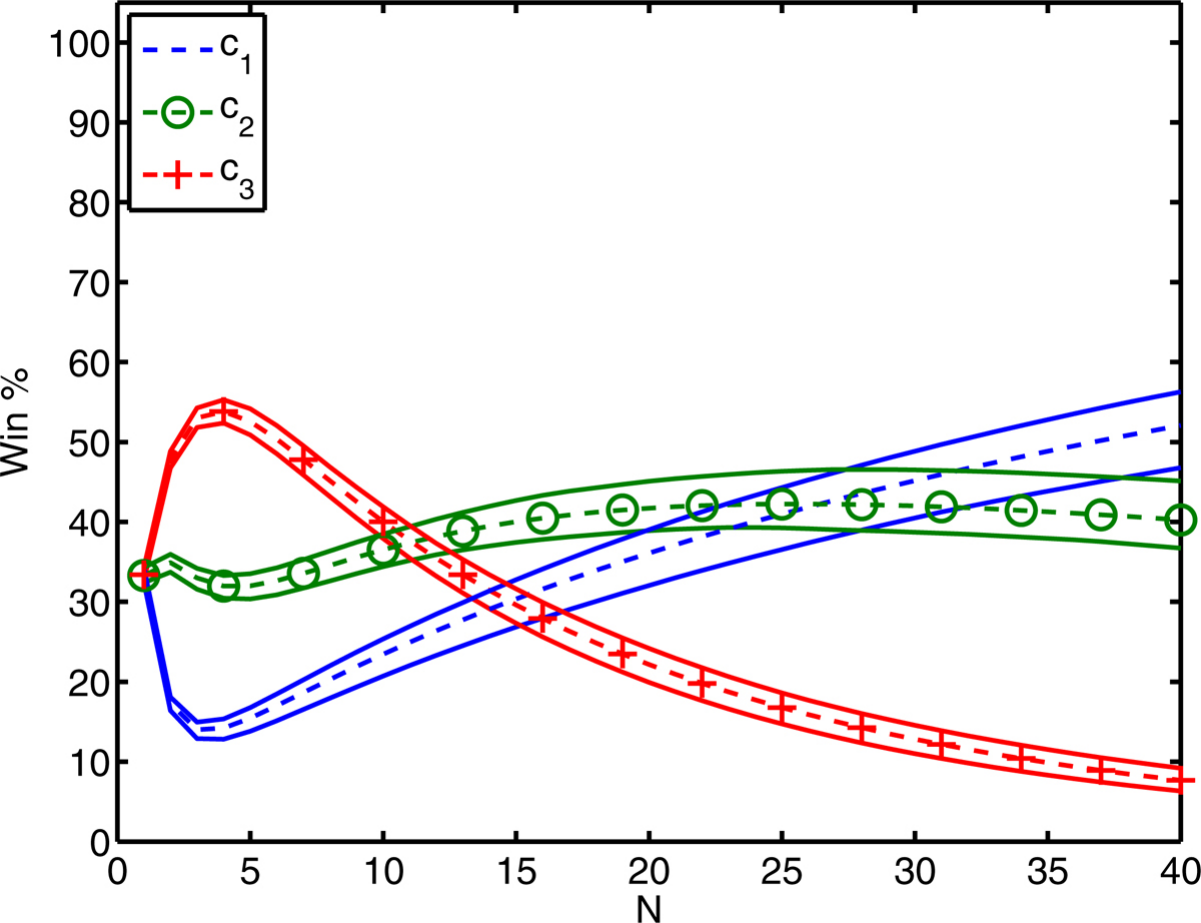
**Win percentage as a function of *N.*** Win percentage for the three candidate classifiers defined in Equation 1. The dotted lines correspond to the exact win percentage for each classifier as a function of the number of randomly selected feature sets, ***N***. The solid lines show the 95% confidence interval for the sampled estimate of win percentage using ***M ***= 10,000 randomly drawn feature sets from the joint density displayed in Figure 2.

Figure 2 plots *p*(*x*,*c*) for the three classifiers. Classifier *c*_3 _clearly performs well for a larger variety of feature sets. However, if we are willing to explore the feature space more thoroughly, classifier *c*_1 _has the longer tail and better chance to win. The dashed lines in Figure 3 plot the win percentage of each hypothetical classifier as a function of the subsample size, *N*. When only one sample is drawn, each classifier has equal chance because their priors are equal. As *N *increases, *c*_3 _has the initial advantage because it has the greater mean performance. For 10<*N*<26, *c*_2 _gains favor because it has moderate mean and variance. However, sampling at least 27 feature sets would suggest using classifier *c*_1 _because it has the best chance of outperforming its peers. The solid lines in Figure 3 plot the 2.5- and 97.5-percentile of 100 iterations of drawing *M *= 10,000 samples from *p*(*x*,*c*) and using the discrete win percentage formula in Equation 11.

This example shows the fundamental difference between classifiers that we are modeling; some classifiers perform well on a wide variety of feature sets, whereas other classifiers perform well on the right set of features. A fair way to compare them is to consider how thoroughly we can explore the feature space given practical computing limitations.

### Synthetic datasets

To explore a wide variety of probability densities, we repeat the previous example 100 times and compare the theoretical and discrete estimate of the win percentage in Equation 7 and 11, respectively. We varied *p*(*x*|*c*) by drawing means from *N*(0.5,0.1), standard deviations from |*N*(0,0.1)|, and *p*(*c*) uniformly. The root mean square error (RMSE) across 100 random distributions, 100 repeated trials of drawing *M *= 1,000 samples, and 1 ≤ *N *≤ 40 was 4.2%. Increasing *M *to 10,000 reduces the error to 1.0%. These results show a clear correspondence between the ideal case with known continuous distributions and the more practical discrete distributions. We next consider cases where the underlying distributions are not parametric.

### Gene expression datasets

We apply the win percentage analysis on clinical gene expression microarray data. We constrain the feature set space to contain all Gaussian feature pairs, corresponding to our Gaussian candidate classifiers. Evaluating all pairs for all datasets required approximately 100 days of computation using MATLAB on 1.95 GHz servers with 20 GB of RAM. We use a discrete distribution of *p*(*x*,*c*) to compute win percentage.

**Figure 4:**
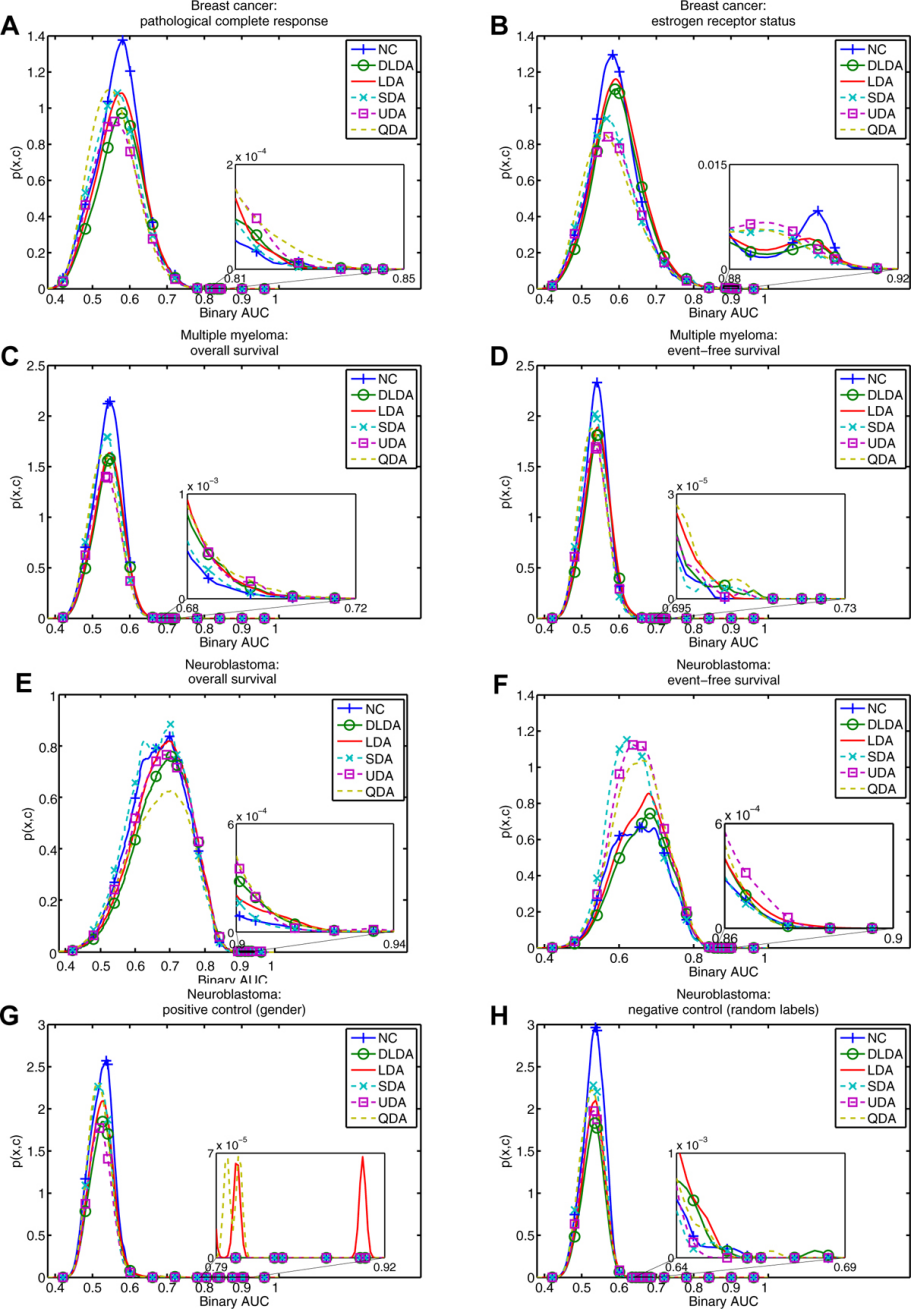
**Kernel smoothed density for FDA MAQC-II data.** The joint probability density functions are plotted for one classifier at a time on the FDA MAQC-II data. The sum of the integrated curves equals one. The window focuses on the better performing feature sets near the extreme of the distribution. The solid lines and dashed lines correspond to linear and nonlinear classifiers, respectively. Panels A - H correspond to the MAQC-II datasets in Table 5.

**Figure 5:**
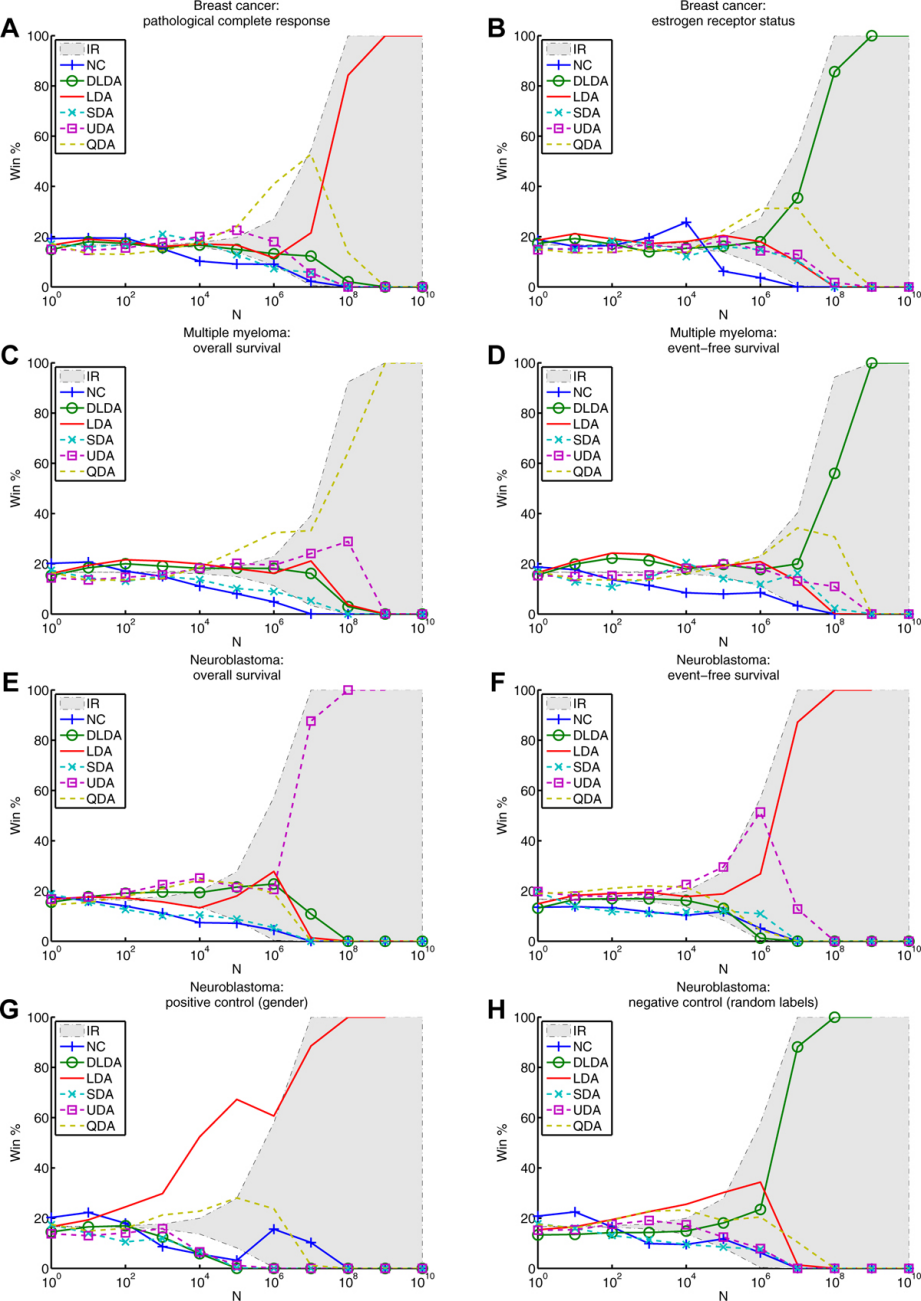
**Win percentage for FDA MAQC-II data.** The exact win percentage is plotted for each classifier as a function of ***N***. For each ***N***, the win percentages sum to one. The solid lines and dashed lines correspond to linear and nonlinear classifiers, respectively. The shaded region corresponds to the 95% confidence interval (CI) for the null distribution of win percentage. Panels A - H correspond to the MAQC-II datasets in Table 5.

Figure 4 shows the distribution of top-classifier performance *p*(*x*,*c*) for each classifier. Figure 5 shows the corresponding win percentage as a function of the number of random feature sets, *N*, for each dataset. For six datasets (Panels A, B, C, D, G and H), nearest centroid wins the largest percentage of all feature sets, illustrated by the largest area under the solid blue density curves, representing *p*(*x*,*c *= NC) in Figure 4; and the tallest solid blue win percentage curve at *N *= 1 in Figure 5. Upon first glance, the conditional distributions would appear somewhat Gaussian but zooming in reveals an unsmooth tail near the peak performance (see insets of Figure 4). In particular, the positive control (panel G of Figure 4) reveals a large disparity in performance between the gender-specific gene set near 0.90 and less specific gene sets near 0.80. The negative control performs much worse in the tail (panel H of Figure 4).

The win percentage at *N *= 10^10 ^reveals the top classifier considering all feature sets. However, these results are not statistically significant because they are based on the performance estimate from only one "best" feature set. Under the null hypothesis that all classifiers have equal chance to perform better, a repeat performance estimate would likely identify a different classifier. Focusing on win percentages outside the shaded statistically insignificant region, we find significant win percentages for smaller *N*. If we are content with a feature set performing among the top 0.05% of all feature sets 99% of the time, we may focus our attention on *N *= 10^4^. Exploring feature sets at this level of thoroughness, UDA performs near the top on five of the six non-control datasets.

For panels A, B, C, D, G, and H linear classifiers appear to perform better when exploring a small number of feature sets. For panels A, B, C, E, F, G, and H nonlinear classifiers perform significantly well for larger *N*. These data suggest nonlinear classifiers perform better when we explore the feature space more thoroughly, and linear classifiers perform better when we do not. On the other hand, the neuroblastoma data in panels E and F show that nonlinear classifiers also have significantly high win percentage for smaller values of *N*. The positive control in panel G shows the most striking result. LDA has significantly high win percentage for *N *≤ 10^5^. Surprisingly, the negative control has statistically significant win percentages for small *N*. This suggests that the null hypothesis that every classifier has equal chance to perform better than its peers on a given feature set is not true. Most striking is *N *= 1. In this case, millions of feature sets are analyzed and the null distribution would expect that every classifier perform better than its peers almost exactly 1/6 of the time. This is clearly not the case, suggesting that knowing the top performing classifier for one feature set may influence our expectation for other feature sets. We revisit this discrepancy in the discussion.

Insignificantly high win percentage helps eliminate some classifiers from consideration. For example for *N *> 1, UDA for dataset B; SDA for dataset C; QDA for dataset D; NC and SDA for dataset E; NC, DLDA, and SDA for dataset F; DLDA, SDA, and for dataset G; and DLDA and SDA for dataset H do not show a significantly high win percentage. Some classifiers clearly fail based on our significance test and may be considered unsuitable for some combinations of dataset and *N*.

### Multiple sampling of microarray data

**Figure 6:**
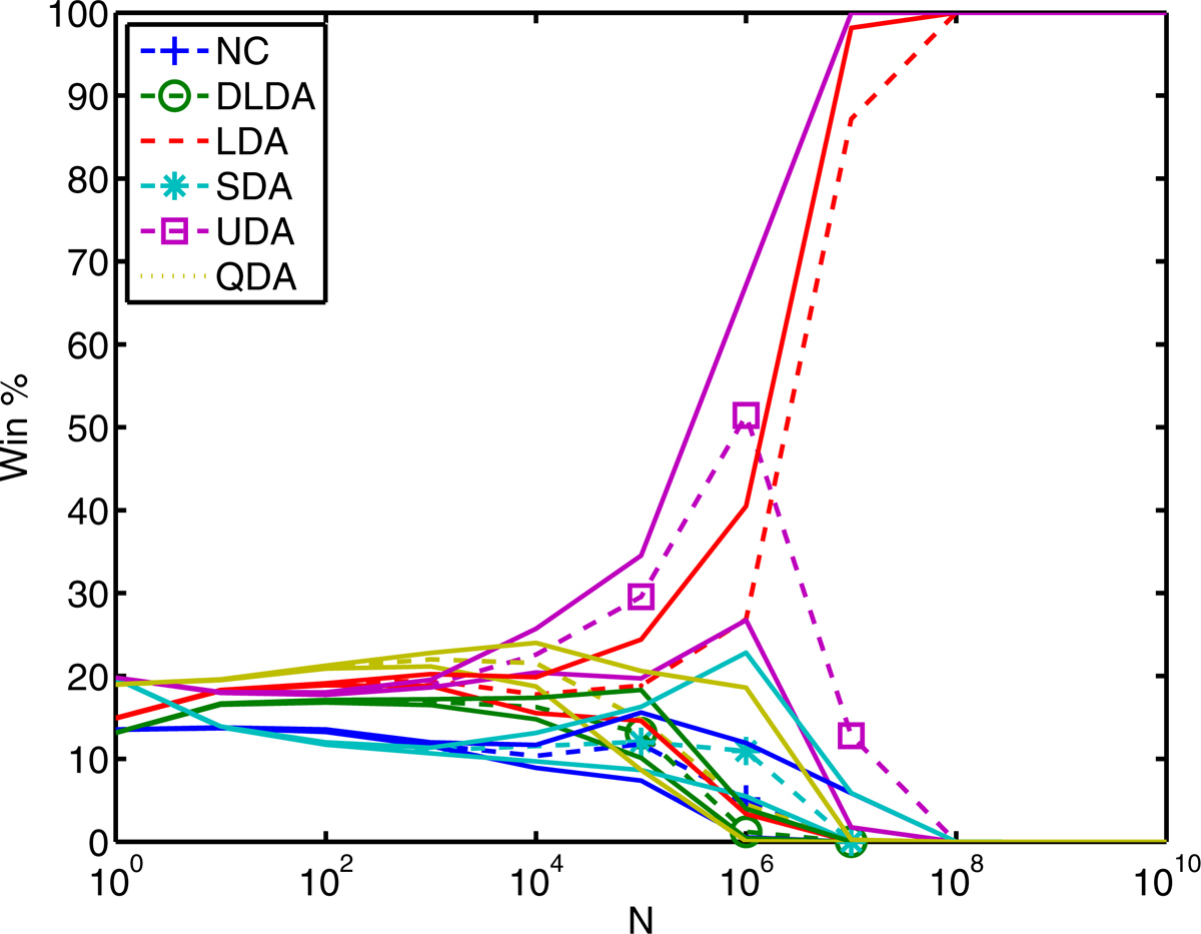
**Confidence intervals for sampled win percentage.** For the neuroblastoma event-free survival datasets, the dashed lines indicate the exact win percentage from panel F of Figure 5. The solid lines indicate the 95% confidence interval computed from 100 trials selecting ***M ***= 10 million feature sets, with replacement.

In the previous example, we used all feature pairs to compute the exact win percentage. However, it will typically be impractical to evaluate all feature sets under consideration. Now, we repeat the previous example using a finite random sample of size *M *from the total number of feature pairs. We repeat 20 trials, each time selecting *M *random samples from all Gaussian feature pairs, and computing the win percentage based on the sample. For a given *M*, variance increases as *N *increases. Figure 6 shows the variation in win percentage for dataset F using *M *= 10 million. The dashed lines are the same as panel F of Figure 5 and the solid lines indicate the 95% confidence interval for the mean performance of the 20 trials. Intuitively, when *N *is much larger than *M*, win percentage depends on a classifier's relative performance on only one (top) feature set. Any variance in that selection transfers to win percentage. For example, some of the trials did not contain the top overall feature set resulting in confusion about the top performing classifier. The confidence interval for UDA and LDA reflects this by spanning the entire range. On the other hand, for smaller *N*, win percentage averages over many feature sets reducing the variance.

**Figure 7:**
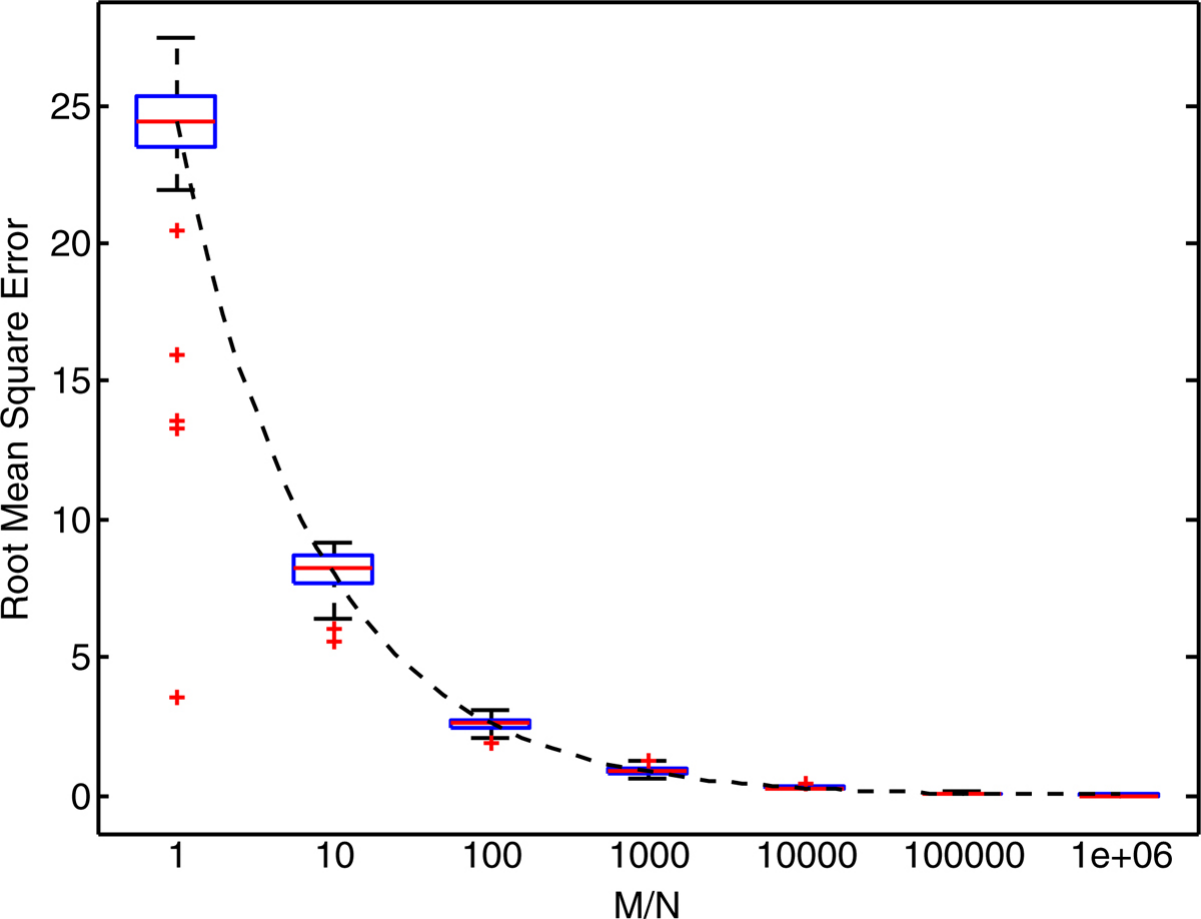
**Root mean square error between exact and sampled win percentage.** Box plots show the distribution of root mean square error as a function of the ratio between ***M ***and ***N***. When the total sample size, ***M***, is large relative to the number of feature sets to explore, ***N***, the error approaches zero. The dashed line indicates the best-fit exponential function (Equation 2) that estimates the error given only ***M ***and ***N***.

Figure 7 reports the root mean square error between the estimated win percentage using *M *samples and the actual win percentage (Figure 5) for all datasets. Interestingly, the ratio between *N *and *M *appears to be the major factor in determining the error in win percentage. Figure 7 shows box plots for RMSE at different ratios of *M *to *N*. For *M *≥ *N*, the RMSE is closely approximated by the following equation:

(2) $$\text{RMSE}\approx 0.24\times {\left(\frac{N}{M}\right)}^{0.48}.$$

The last row in Table 2 shows the predicted RMSE based on Equation 2. In order to accomplish a RMSE of less than 1%, these data suggest selecting *M *> 750*N*.

**Table 2:** Random features required for *ε *and *p*

*N*	*ε*	*p*	*ε*	*p*
1	0.001	0.999	1 × 10^-6^	1.000
10	0.001	0.499	1 × 10^-6^	0.749
100	0.001	0.0667	1 × 10^-6^	0.129
1K	0.001	0.00688	1 × 10^-6^	0.0137
10K	0.001	0.000691	1 × 10^-6^	0.00138
>10K	0.001	≈ 6.91/*N*	1 × 10^-6^	≈ 13.8/*N*

The fraction of top feature sets, *p*, gets smaller as *N *increases. For *p *near zero, *p * ≈ -(ln *ε*)/*N *corresponding to the last column of the table.

## Discussion

The suitability of a classifier for a dataset cannot be determined after feature selection. We show that given the right feature set, any of the six classifiers examined here could be judged as suitable. However, if we consider the difficulty of finding a good feature set for a classifier we may evaluate a classifier for a dataset rather than for a particular feature set. These ideas motivate our proposed win percentage measure for comparing the relative suitability of a classifier to a dataset. However, as an initial investigation there are several points that bear consideration for future study.

We provide examples to illustrate the potential usefulness of win percentage for analyzing and comparing classifier performance. Eventually, we would like to use win percentage to inform the model building process. One approach that seems promising is to use win percentage to assist practitioners in selecting or eliminating classifiers from consideration. After determining a suitable classifier, we could choose a tailored feature selection method within cross-validation to estimate its performance.

One key aspect of our approach is that we do not attempt to model the absolute performance of each classifier across the feature space. Win percentage only compares classifiers and does not comment on their absolute performance. In general, we would expect these classifiers to perform near chance on the random labels. However, we observe that the mean of *X *appears to exceed 0.5 on every dataset including the negative control. This bias can be explained by the selection of one best performance among the six candidate classifiers. The expected value of the largest sample among six random samples from a Gaussian distribution is *μ *+ 1.27*σ *[14,16]. Therefore, it is reasonable to expect the observed mean shifts. Future work might incorporate whether the top classifier performs better than chance on the dataset.

In estimating the performance of each classifier for a feature set, we use two iterations of three-fold cross-validation. Such a method is itself a randomized algorithm and multiple trials produce different results. In particular, our notion of "best" may be extended to include those classifiers that perform insignificantly differently from the best or "among the better" classifiers during cross-validation. This improvement would likely move all win percentage curves closer to 1/6 in Figure 5 and reduce the apparent significance of all results. In particular, it would partly address the apparent significance of the negative control (randomly labeled) dataset.

Intuitively, we would expect the negative control to exhibit win percentages that are likely to be drawn from the null distribution. For *N *= 1 in panel H of Figure 5, this is obviously not the case. Another contributing factor could be that the feature sets are not independent of each other. If a classifier performs better than its peers on a single feature, it would stand to reason that it is more likely to perform better than its peers on *all *feature sets containing that feature. If this is the case, it reduces the number of independent observations used to compute the null distribution. In the extreme, the win percentage computed from individual features is also exhibited by all feature pairs. In this case, the number of independent observations is reduced from *C*(*F*,2) combinations to merely *F*. We can easily adjust our critical win percentages by reducing *M *to *F *in Equation 10 and using Equations 12-14. By doing this, none of the win percentages for the random endpoint are significant. However, estimating the actual redundancy among feature sets for an arbitrary dataset proves difficult as does adjusting *M*. Future work could estimate the null distribution empirically by computing win percentage using multiple permutations of the class labels for each dataset. This computationally expensive approach could lend insight into the true null distribution and the effective number of independent feature sets implied by *M *in our theoretical null distribution.

Although we focused on a pair-wise analysis of the feature space, our proposed approach easily extends to higher dimensions. Whereas it is often impractical to estimate the performance of all feature triplets or quadruplets, these data suggest that sampling only 750*N *of these higher dimensional feature sets may be useful in comparing classifiers that explore *N *random feature sets. As the feature sets become larger, it may also be useful to define the probability of selecting each feature set. For example, one can favor features based on a preferred ranking criterion. Whereas heuristic methods quickly find local minima, the randomness in this approach makes a more thorough exploration of the feature space possible.

## Conclusions

We propose a novel way to compare classifiers based on the probability that they will outperform their peers (win percentage) on a random sample of the feature sets. Unlike cross-validation that estimates classifier *performance *using random subsets of all samples, win percentage estimates classifier *suitability *using random subsets of all feature sets. First, we illustrate the utility of this approach using all Gaussian feature pairs. Then, we show that precise estimates (within 1%) can be achieved using a smaller random sample of all feature pairs.

We show that win percentage performs as expected on synthetic datasets and then apply it to real microarray data. We observe that the selection of the most suitable classifier does not only depend on the dataset but also on the thoroughness of feature selection. In addition, the results suggest that nonlinear classifiers perform better when the feature space is explored more thoroughly and linear classifiers perform better when it is not. Using a theoretical null distribution, we can exclude some classifiers from consideration because their win percentage falls within a statistically insignificant region.

## Methods

In an effort to assess the suitability of a classifier to a dataset, we first attempt to find feature sets for which each classifier performs better than its peers. In order to compare multiple classifiers across all feature sets, we propose estimating the probability that each will perform better than its peers will, given an incomplete sample of feature sets. We refer to this probability as "win percentage." If a classifier performs well on one feature set and poorly on all others, the likelihood of winning will depend on the certainty in selecting that feature set. However, a classifier that performs well on a large variety of feature sets is more likely to win even when only a small group of feature sets is considered.

### Randomized feature selection

As an analytical tool to define classifier suitability to a dataset, we specify a wrapper-based Monte Carlo feature selection method, MCW (shown in Table 3) that draws a fixed number of feature sets randomly and evaluates each using candidate classifiers. The function 'randomSubset' uses a pseudorandom number generator to select a random feature set from among the total number of feature sets under consideration with replacement. For example, *F *features have 2*^F ^*total subsets or *C*(*F*,2) combinations of feature pairs. In practice, the user could favor some subsets by defining the probability of selecting each subset. For example, one might specify a prior probability on the number of features in a feature set, *p*(*N_F_*). The 'performance' function used for MCW returns the highest estimated performance among all candidate classifiers along with the labels of the top performing classifiers in the set *C_i_*. If that feature set returns the top performance so far, one classifier *c*_out _is chosen at random.

**Table 3:** Pseudocode for a Monte Carlo wrapper-based feature selection algorithm

MCW(*S*, *N*)
1. *x*_out _= -∞
2. For *i *= 1 to *N*
*S_i _*= randomSubset(*S*)
(*x_i_*, *C_i_*) = performance(*S_i_*)
If *x_i _*>*x*_out_,
*S*_out _= *S_i_*, *x*_out _= *x_i_*, *c*_out _= randomElement(*C_i_*)
3. output *S*_out_, *x*_out_, *c*_out_

The input, *S*, is the set of all features, and *N *is the total number of feature subsets to draw randomly. The variable *x_i _*is the performance of the top classifier for subset *S_i_*, and *C_i _*is the label of the top classifier. *S*_out_, *x*_out_, and *c*_out_, return the top performing feature set, top estimated performance, and top classifier, respectively.

### Selecting the number of iterations for MCW

Because it is impractical to explore all possible subsets to find the optimal feature set for each classifier, we choose the number of evaluated feature sets, *N*, to balance practical computing constraints and tolerated bounds on feature set performance. Specifically, we introduce the desired fraction, *p*, of top-performing feature sets among which MCW's selected feature set will likely belong. The probability that MCW's selected feature set is *not *among the top *p *percent of top-performing models is *ε*, the failure tolerance. The probability that a random feature set rates in the top *p *percent of all feature sets is *p*. That is, a random feature set has a 50% chance to be in the top 50% and a 1% chance to be in the top 1%. As we evaluate more feature sets, the chance of failure decreases such that:

(3) $$\text{Pr}\left(\text{Failure}\right)=\varepsilon ={\left(1-p\right)}^{N}.$$

Given a tolerated chance of failure, *ε*, and the desired top fraction of all feature sets, *p*, we solve for the number of necessary iterations of the algorithm:

(4) $$N=\frac{\text{ln}\varepsilon }{\text{ln}\left(1-p\right)}.$$

Table 2 provides an example of how the fraction of top feature sets, *p*, gets smaller as we increase *N*. For *p *near zero, *p *can be approximated as the following:

(5) $$\begin{array}{llll} p & =1-\varepsilon^{1/N}\; & & \;\\ \text{ln}\left(1-p\right) & =\frac{\text{ln}\varepsilon }{N}\; & & \;\\ p & \approx \frac{-\text{ln}\varepsilon }{N},\; & & \;\\ & \; & \end{array}$$

where we use the first-order Maclaurin approximation for ln(1-*p*)≈-*p *. This indicates a simple inverse relationship between number of random samples and size of the fraction of top feature sets.

### Theoretical win percentage

Now that we have specified a classifier-agnostic feature selection method, we may derive a suitable criterion to compare different classifiers considering the uncertainty in feature selection. First, we consider the probability density of the best performance of all candidate classifiers, *x_i_*, computed at each iteration of MCW. For now, we ignore which classifier produces a feature set's performance and treat it as a random variable, *X*, with probability density function *p*(*x*). The probability that a particular performance, *x*, will be the best in a random sample of size *N *is the following [17]:

(6) $$p\left(x=\text{max}\left(x_{1}\ldots x_{N}\right)\right)=N\cdot P{\left(X<x\right)}^{N-1}p\left(x\right),$$

where *P *is the cumulative density function and the *x_i _*are drawn from *p*(*x*). In terms of MCW, *p*(*x *= max(...)) is the probability density function of the output variable *x*_out_. Although we could approximate this distribution by running MCW a large number of times, Equation 6 represents the exact density considering all possible MCW results. When comparing classifiers, we are more interested in which classifier performs better at each *x*. Therefore, we consider the joint distribution *p*(*x*,*c*), where *c *is a categorical random variable representing the classifier that performs best for a feature set with performance *x*. The probability that classifier *c *performs best in a random sample of size *N *is the following:

(7) $$\text{win}\left(c\right)=\overset{\infty }{\underset{-\infty }{\int }}p\left(c|x\right)p\left(x=\text{max}\left(x_{1}\ldots x_{N}\right)\right)dx,$$

where the integrand is the probability that classifier *c *performs best for a feature set with performance *x*. In terms of MCW, win(*c*) is the probability that MCW will output *c*_out _= *c*. Given a reasonable approximation to *p*(*x*,*c*) we can estimate which classifiers are more likely to win without needing to run MCW.

### Discrete win percentage

When the distribution of performance for each classifier cannot be reasonably approximated by a Gaussian or other parametric distribution, we model *X *as a discrete random variable. We estimate *p*(*x*,*c*) from a sufficiently large set of random samples,

(8) $$S_{M}=\left\{\left(x_{i},c_{i}\right)|1\leq i\leq M\right\}.$$

**Figure 8:**
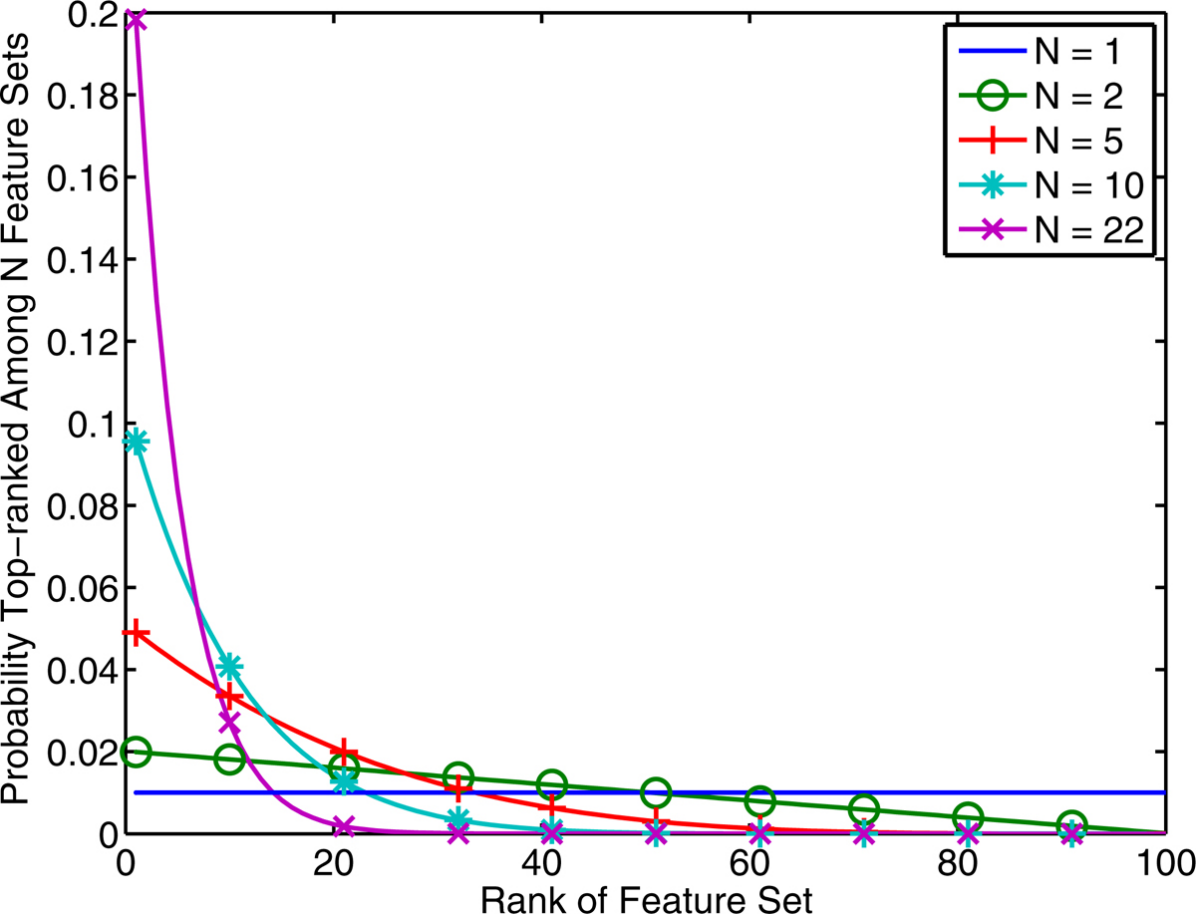
**Selection pressure as a function of *N.*** Each plot shows the probability mass function for feature sets ranked by estimated performance. We consider a total of ***M ***= 100 unique feature sets each with a corresponding estimated performance. The five line plots show the probability that each of the ***M ***feature sets will be selected as best in a random selection of ***N ***sets, with replacement. As ***N ***increases, the feature sets with higher performance (lower rank) are more likely to be selected.

We then draw hypothetical subsets of *S_M _*within MCW:

(9) $$S_{N}=\left\{\left(x_{s_{i}},c_{s_{i}}\right)|1\leq i\leq N\right\},$$

to derive the win percentage for a random sample of size N:

(10) $$\begin{array}{c} w_{rank\left(x\right)}=p\left(x=\text{max}\left(x_{s_{1}}\ldots x_{s_{N}}\right)\right)=\\ \;{\left(\frac{M-rank\left(x\right)+1}{M}\right)}^{N}-{\left(\frac{M-rank\left(x\right)+1-count\left(x\right)}{M}\right)}^{N}, \end{array}$$

where 'rank' is the rank of *x *among all *M *samples in descending order and 'count' is the number of samples with performance *x *in *S_M_*. The first fraction is the probability of randomly selecting with replacement *N *feature sets all of which have the same rank as *x *or worse. The second fraction removes those that do not contain a sample with performance *x*. Notice that for *x *to hold the maximum performance, it must be among the *M *samples. Figure 8 plots this distribution as a function of the rank of *x *for *M *= 100. If we select one random sample, each sample has equal probability of being the best regardless of rank. As *N *increases, the probability shifts toward the better ranked feature sets, until at the limit only the best feature set is likely to be selected. Selection pressure refers to an analogous concept for genetic algorithms [17].

Estimating which classifier is more likely to perform best given the number of randomly sampled feature sets *N *involves a simple summation over all *M *samples, (*x_i_*,*c_i_*):

(11) $$\text{win}\left(c\right)=\overset{M}{\underset{i=1}{\sum }}\left\{\begin{array}{ccc} \frac{p\left(x_{i}=\text{max}\left(x_{s_{1}}\ldots x_{s_{N}}\right)\right)}{\left|C_{i}\right|} & , & c\in C_{i}\\ 0 & , & \text{otherwise} \end{array}\right).$$

Again, win(*c*) is the probability that MWC will output *c*_out _= *c *and *C_i _*is the set of classifiers with equal performance, *x_i_*. Win percentage provides the exact fraction of times classifier *c *performs better than its peers among all possible subsets *S_N _*used by MCW.

Although other randomized algorithms could be used to estimate a related definition of win percentage, we chose MCW because of its simplicity. For example, a Las Vegas algorithm continues to explore feature sets until a convergence criterion is met [12], resulting in an unpredictable *N *and complicating the resulting mathematical formulation.

### Statistical significance of win percentage

Win percentage provides a statistic that potentially reveals which classifiers are more or less suited to a particular problem. We introduce a method for significance testing that helps determine which values for win percentage are unlikely to occur by chance. Specifically, we estimate the null distribution for win percentage and use it to calculate a p-value for each classifier's win percentage.

Under the null hypothesis, each classifier has an equal chance to perform better than its peers for a given feature set. For a single classifier, win percentage depends on which feature sets the classifier wins. We model winning and losing as a Bernoulli process where the classifier of interest has a 1/6 chance to win each feature set and a 5/6 chance to lose. Ignoring ties, win percentage is a weighted average of Bernoulli random variables:

(12) $$\text{win}\left(c\right)=\overset{M}{\underset{i=1}{\sum }}w_{i}b_{i},$$

where *w_i _*is the probability defined in Equation 10 and *b_i _*is a Bernoulli random variable equal to one when the classifier wins the *i*th-ranked feature set. Therefore, win percentage is a random variable with the following mean and variance:

(13) $$\begin{array}{c} E\left[\text{win}\left(c\right)\right]=\overset{M}{\underset{i=1}{\sum }}w_{i}E\left[b_{i}\right]=q\overset{M}{\underset{i=1}{\sum }}w_{i}=q\\ Var\left[\text{win}\left(c\right)\right]=\overset{M}{\underset{i=1}{\sum }}w_{i}^{2}Var\left[b_{i}\right]=q\left(1-q\right)\overset{M}{\underset{i=1}{\sum }}w_{i}^{2}. \end{array}$$

We model the null distribution for win percentage as a beta distribution with a mean of 1/6 and a variance determined by *M *and *N*. Specifically, we use the method-of-moments to estimate the parameters of the beta distribution:

(14) $$\alpha =q\left(\frac{1}{\sum_{i=1}^{M}w_{i}^{2}}-1\right),\;\beta =\left(1-q\right)\left(\frac{1}{\sum_{i=1}^{M}w_{i}^{2}}-1\right).$$

Using a desired false positive rate of 5%, we use a Bonferroni adjusted significance level of 0.01 (five degrees of freedom for six classifiers). Then, we compute the critical win percentages at the 0.5^th ^and 99.5^th ^percentile of the null distribution. We consider the win percentages between the two critical values to be statistically insignificant.

### Classifiers and performance metric

Given a dataset of features and labeled samples, we estimate the distribution of best classifier performance using cross-validation. Each feature set produces one sample (*x_i_*,*C_i_*), where *x_i _*is the maximum cross-validation performance among candidate classifiers and *C_i _*is the set of classifiers achieving the highest performance. Although a number of methods for cross-validation could be used, we chose two iterations of three-fold cross-validation for its efficiency compared to more iterations or more folds. This allows us to explore a much larger feature space than more computationally complex performance estimates.

In order to compare a variety of linear and nonlinear classifiers without needing computationally expensive parameter selection, we focus on six Gaussian Bayes classifiers. Specifically, we represent each class as a multivariate Gaussian distribution with a possibly constrained covariance matrix. We classify new samples with the label associated with the most likely class distribution using uniform priors. The covariance matrix is either constrained to be the same for both classes (pooled) or allowed to vary between classes (unpooled). In addition, the covariance is proportional to the identity matrix (spherical), uncorrelated (diagonal), or unconstrained (full). Table 4 summarizes the six classifiers. In particular, the well known nearest centroid classifier corresponds to pooled spherical covariance, linear discriminant analysis corresponds to pooled full covariance, and quadratic discriminant analysis corresponds to unpooled full covariance. Classifiers utilizing pooled covariance are linear in that they construct a linear decision boundary, whereas those that compute covariance independently for each class are nonlinear and construct a quadratic decision boundary. Classifier complexity increases when you move down or to the right in the table. The degrees of freedom for two-class two-dimensional data are listed in parentheses.

**Table 4:** Relationships between candidate classifiers

Covariance type	Spherical	Diagonal	Full
Pooled	Nearest centroid (NC, 5 d.f.)	Diagonal linear discriminant analysis(DLDA, 6 d.f.)	Linear discriminant analysis (LDA, 7 d.f.)
Unpooled	Spherical discriminant analysis (SDA, 6 d.f.)	Uncorrelated discriminant analysis (UDA, 8 d.f.)	Quadratic discriminant analysis (QDA, 10 d.f.)

When comparing classifier performance, the fraction of correctly classified test samples (accuracy) is a very common performance metric. Usually, the samples for training and testing are selected to have equal proportions in each class. In this work, we use the average of sensitivity and specificity, also known as binary AUC (area under the receiver operating characteristic curve using binary labels). When the class proportions are equal, this measure is equivalent to accuracy. However, in many biomedical applications including those in this paper, the class proportions are skewed. For these data, the average of sensitivity and specificity represents a class-balanced accuracy, *i.e*., the expected accuracy if the class proportions were balanced. In practical applications of biomedical classification, it may be desirable to favor sensitivity over specificity (or vice versa), justifying a weighted average of the two.

### Datasets and feature space

We analyze eight classification problems from three cancer datasets from the FDA MicroArray Quality Control Phase-II Project (MAQC-II) [15]. Table 5 summarizes eight datasets from MAQC-II. The breast cancer dataset [18] originates from microarray data collected from fine needle aspiration specimens from newly diagnosed patients before treatment. Dataset A classifies each patient as either having pathological complete response or residual invasive cancer after preoperative chemotherapy. Dataset B classifies patients based on estrogen receptor status as determined by immunohistochemistry. The multiple myeloma dataset [19] originates from microarray data collected from bone marrow plasma cells in newly diagnosed patients, and classifies them based on overall survival (dataset C) and event-free survival (dataset D) using a 730-day cutoff. The neuroblastoma dataset [20] originates from microarray data collected from newly diagnosed patients and classifies them based on overall survival (dataset E) and event-free survival (dataset F) using a 900-day cutoff. In addition, we analyze a positive control labeled by patient gender (dataset G) and a negative control labeled randomly (dataset H) using the same neuroblastoma patients.

**Table 5:** Dataset properties

ID	Description	Total features	Gaussian features	Gaussian pairs
A	Breast cancer, pathological complete response	22283	10404	5.4 × 10^7^
B	Breast cancer, estrogen receptor status	22283	10154	5.2 × 10^7^
C	Multiple myeloma, overall survival	54675	16736	1.4 × 10^8^
D	Multiple myeloma, event-free survival	54675	16147	1.3 × 10^8^
E	Neuroblastoma, overall survival	10707	3095	4.8 × 10^6^
F	Neuroblastoma, event-free survival	10707	3122	4.9 × 10^6^
G	Neuroblastoma, positive control (gender)	10707	3050	4.6 × 10^6^
H	Neuroblastoma, negative control (random)	10707	3064	4.7 × 10^6^

Although our approach is extensible to any probabilistic sampling of the feature space, for this investigation we limit ourselves to feature sets containing exactly two features. This allows us to compute an exact win percentage using the complete feature space and compare it to win percentage computed using only a fraction of the complete space. We could equally apply this methodology to sets of three or more features; however, we believe feature pairs illustrate the principal. We explore all features that exhibit a prescribed level of normality based on the standard error of the kurtosis of each feature. Thus, we explore the complete feature space of Gaussian feature pairs. Table 5 also lists the number of features passing the Gaussian test for each dataset and the total number of feature pairs evaluated.

### Illustrating the utility of each classifier for each dataset

We propose win percentage as a way to assess the suitability of a classifier to a dataset. If the choice were obvious, there would be no need for such a measure. To illustrate the utility of each classifier for each datasets, we attempt to find gene pairs for which each classifier performs statistically significantly better than its peers. We use the results of the coarse cross-validation (two iterations of three-fold), to rank feature sets by top classifier performance. Then, we reanalyze the top 100 feature sets using a finer-grained 20 iterations of five-fold cross-validation in order to more precisely estimate performance and to find statistically significant differences. Because we use the same folds for every classifier, we use a paired *t*-test to compare the mean performance of the top classifier to each of the remaining classifiers. For each classifier, we select one feature set that demonstrates its utility, provide a scatter plot to show how the classifier fits the data, and report performance results as well as significance.

### Sampling with replacement

In this work, we chose to draw random samples with replacement. Alternatively, we could draw randomly without replacement so that no feature set is drawn more than once within MCW. Although this is a slightly more efficient way to explore the feature space, sampling with replacement allows a simpler presentation and mathematical representation. The difference is subtle even when randomly sampling a subset of size *M *from a total set of size *M*. In this case, the expected fraction of unique samples is 63.2% [21]. Using MCW, we typically draw subsets much smaller than the total feature space. The expected fraction of unique samples in a subset of size *N *from a set of size *M *is (1-(1-1/*M*)*^N^*)*M*/*N*. Conservative estimates of *M *= 1000000 and *N *= 100000 result in 95% unique samples, suggesting that we would need to draw ~5% more samples to achieve the same feature space coverage as a random sample without replacement.

## Competing interests

The authors declare that they have no competing interests.

## Authors' contributions

RMP conceived of win percentage as a way to compare classifiers, designed the study, and drafted the document. JHP helped implement the classifiers, revise the document, and test significance. MDW initiated the microarray quality control and high-throughput bio-molecular data mining investigation from which the idea for win percentage spawned, acquired funding to sponsor this effort, and directed the win percentage project and publication. All authors read and approved the final manuscript.

## Endnote

^a ^This work is based on an earlier work: Win percentage: A novel measure for assessing the suitability of machine classifiers for biological problems, in ACM International Conference on Bioinformatics and Computational Biology, (Aug. 1-3, 2011) ^© ^ACM, 2011.

## Supplementary Material

Additional file 1 Please see additional file: File01_additional.pdf for supplemental material that contains classifier discrimination plots and classifier performance for pair-wise analysis on all cancer datasets. Click here for file
