# Supplementary material for: Win percentage: a novel measure for assessing the suitability of machine classifiers for biological problems
Source: BMC Bioinformatics. 2012 Mar 21;13(Suppl 3):S7. doi: 10.1186/1471-2105-13-S3-S7 (PMC3485616; doi:10.1186/1471-2105-13-S3-S7)
Supplement: Additional file 1 — Please see additional file: File01_additional.pdf for supplemental material that contains classifier discrimination plots and classifier performance for pair-wise analysis on all cancer datasets. [file 1471-2105-13-S3-S7-S1.pdf]

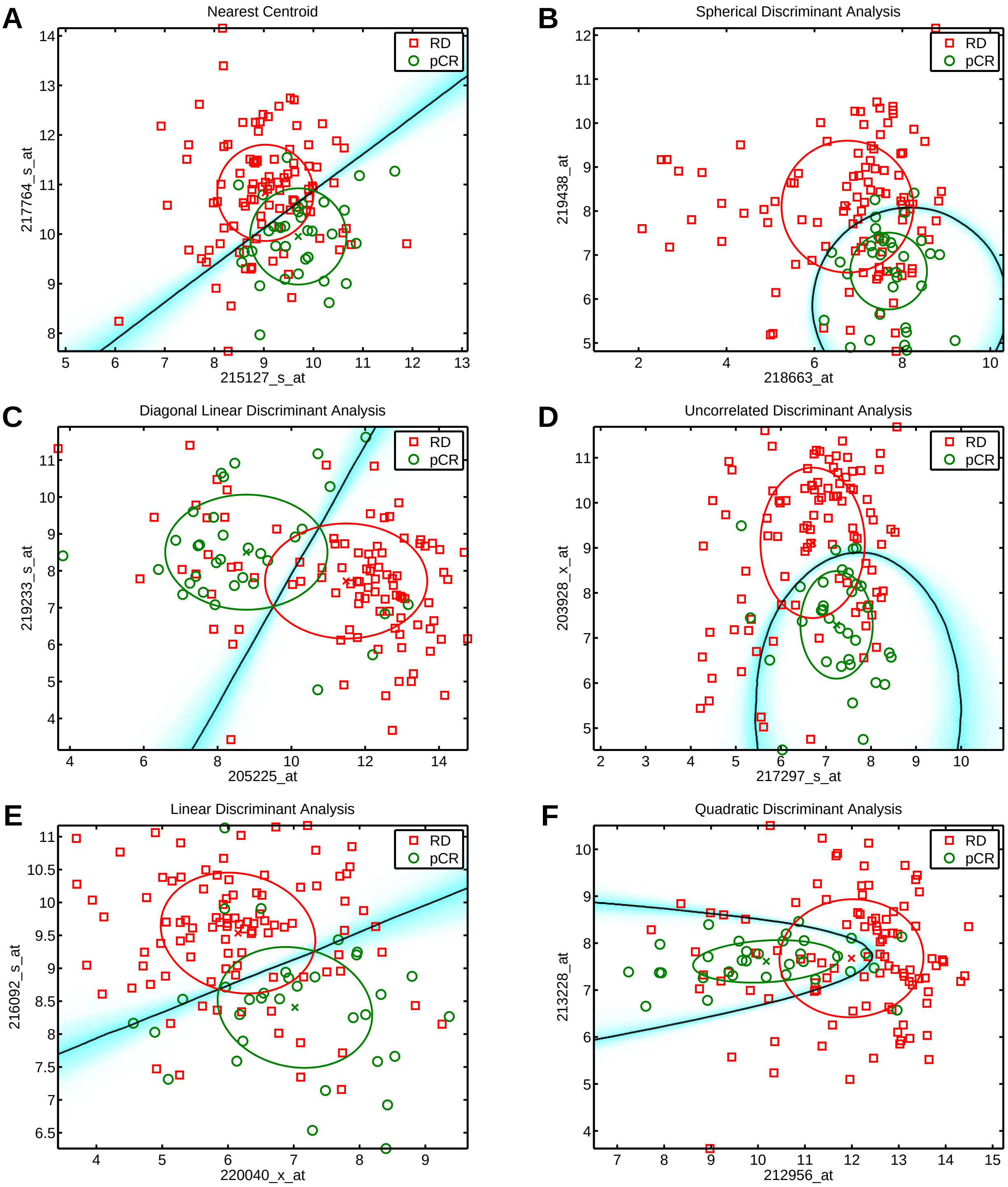

Figure S1 – **Classifier discrimination plots for breast cancer, pathological complete response. RD indicates residual invasive tumor, and pCR indicates pathological complete response.**

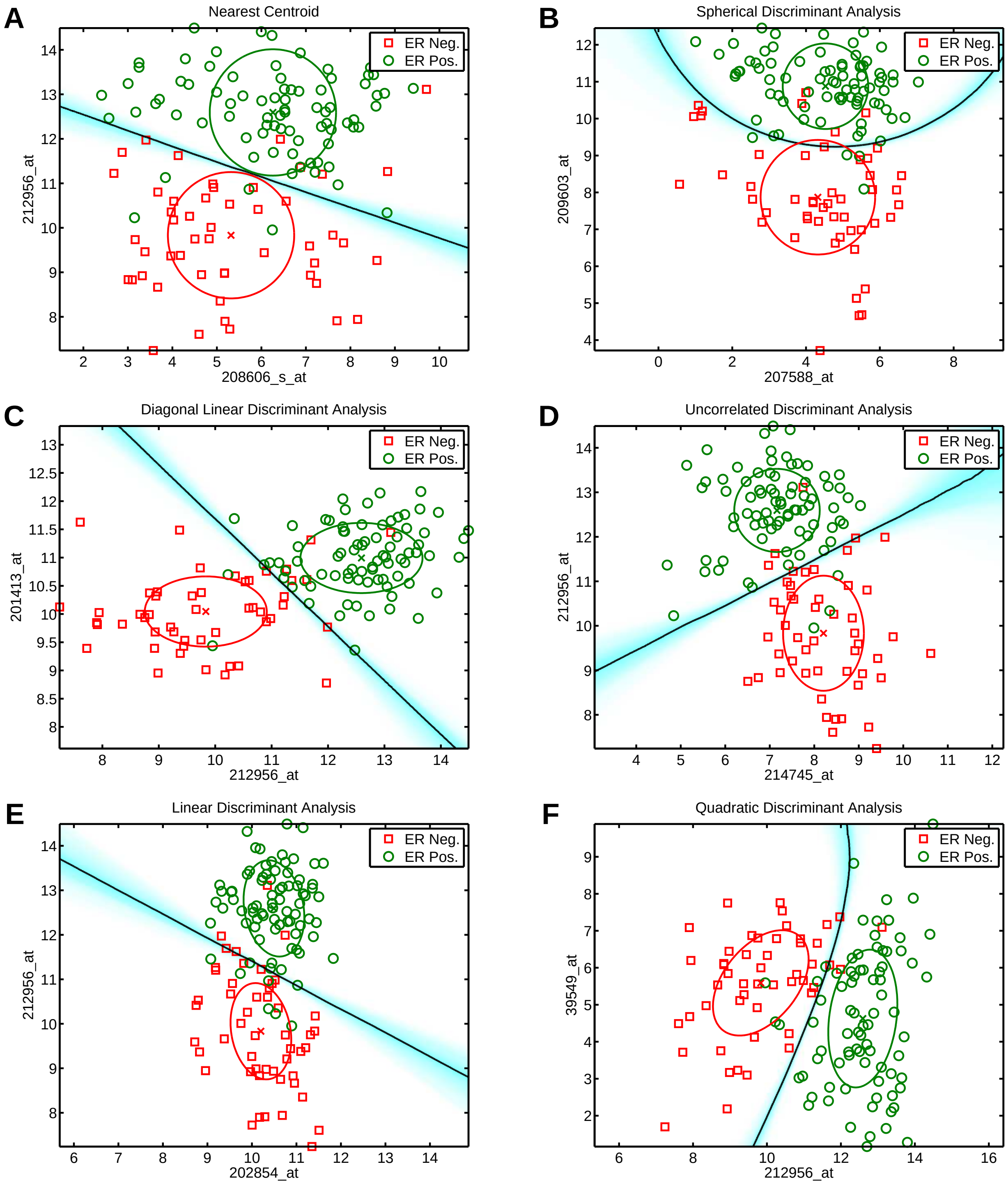

Figure S2 – **Classifier discrimination plots for breast cancer, estrogen receptor status. ER pos. and ER neg. refers to patients that have over-expressed estrogen receptors based on immunohistochemistry or the lack thereof, respectively.**

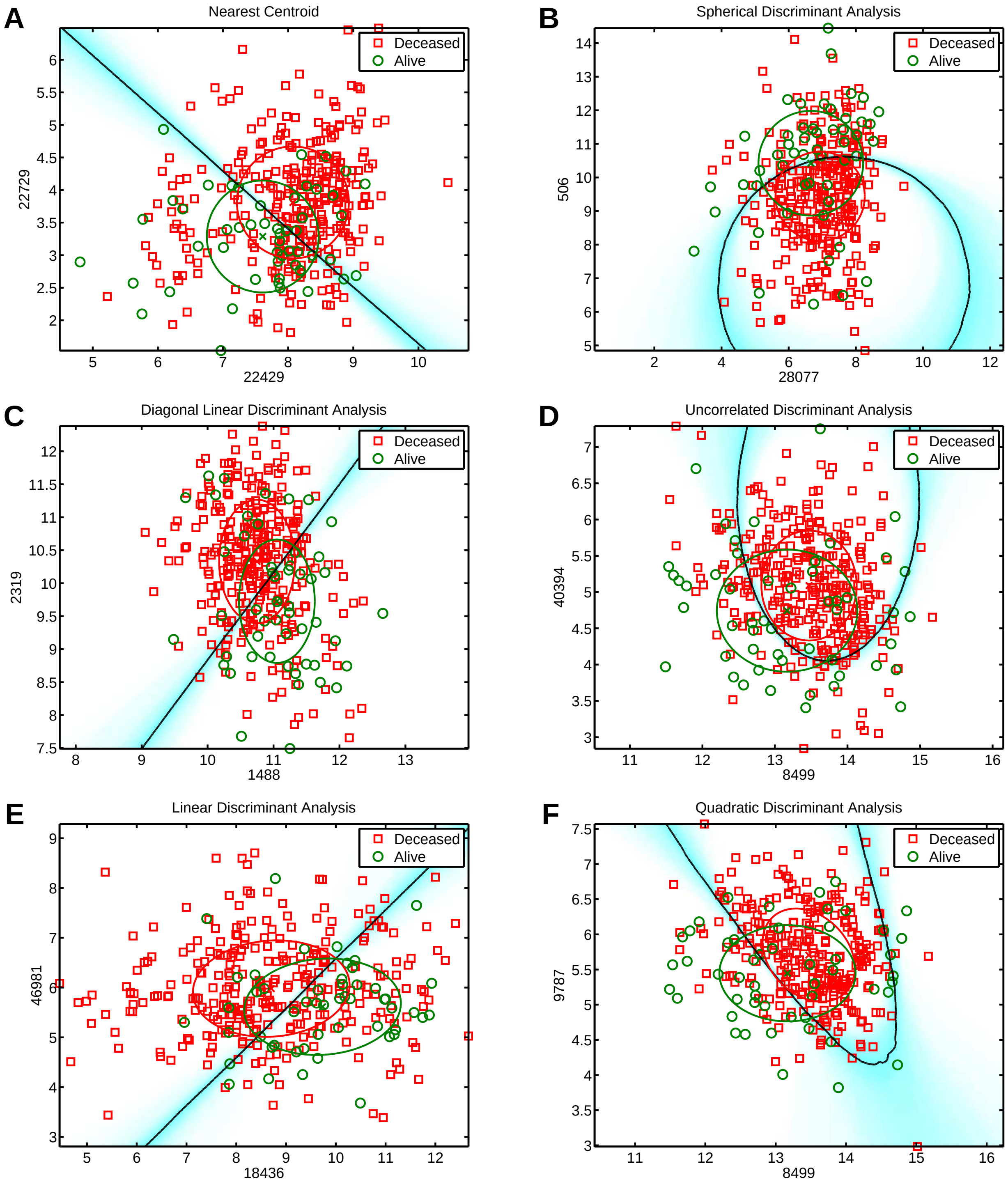

Figure S3 – Classifier discrimination plots for multiple myeloma, overall survival. Labels indicate whether or not the patient survived 730 days after diagnosis.

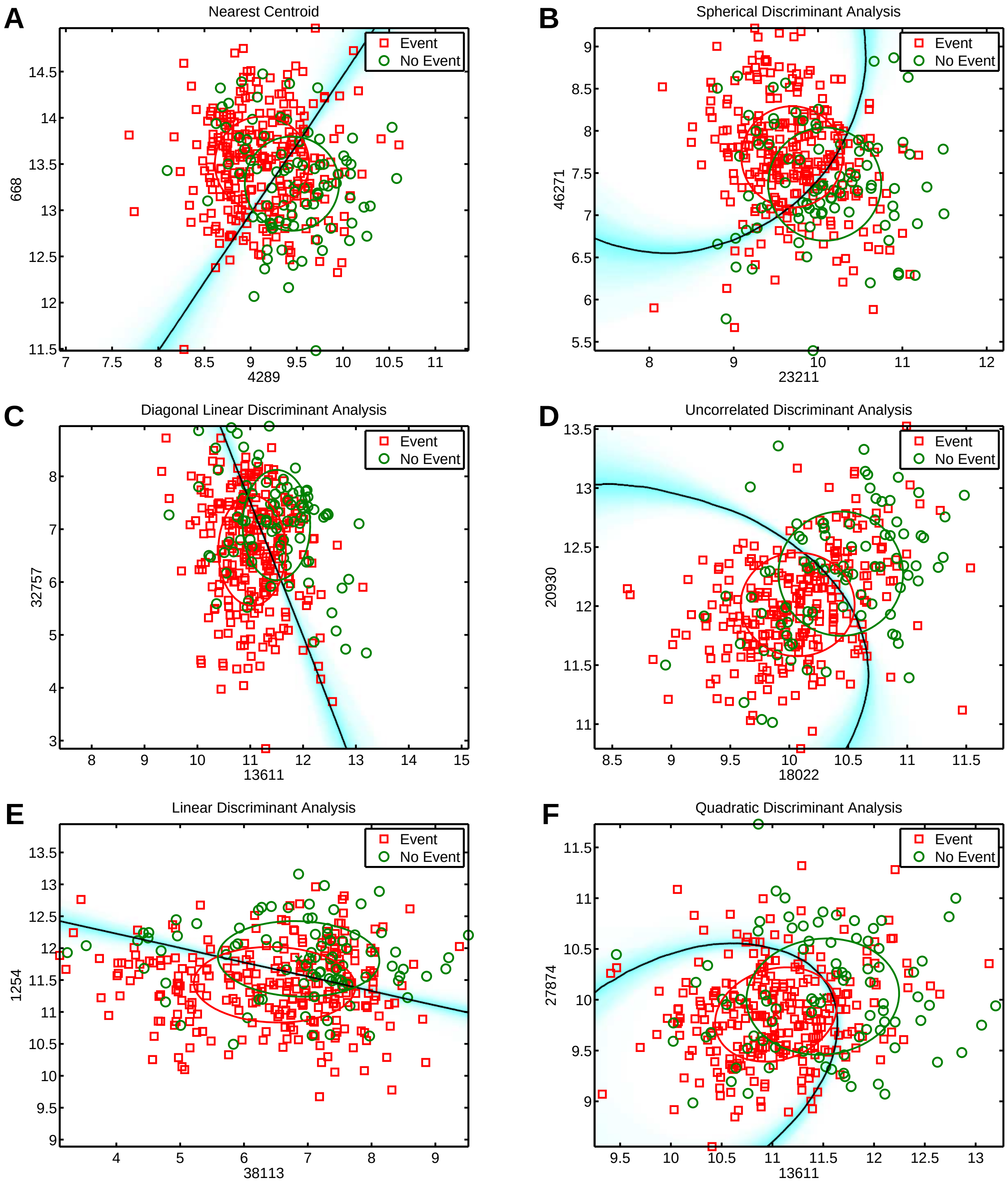

Figure S4 – Classifier discrimination plots for multiple myeloma, event-free survival. Labels indicate whether or not the patient survived without a medical event for 730 days after diagnosis.

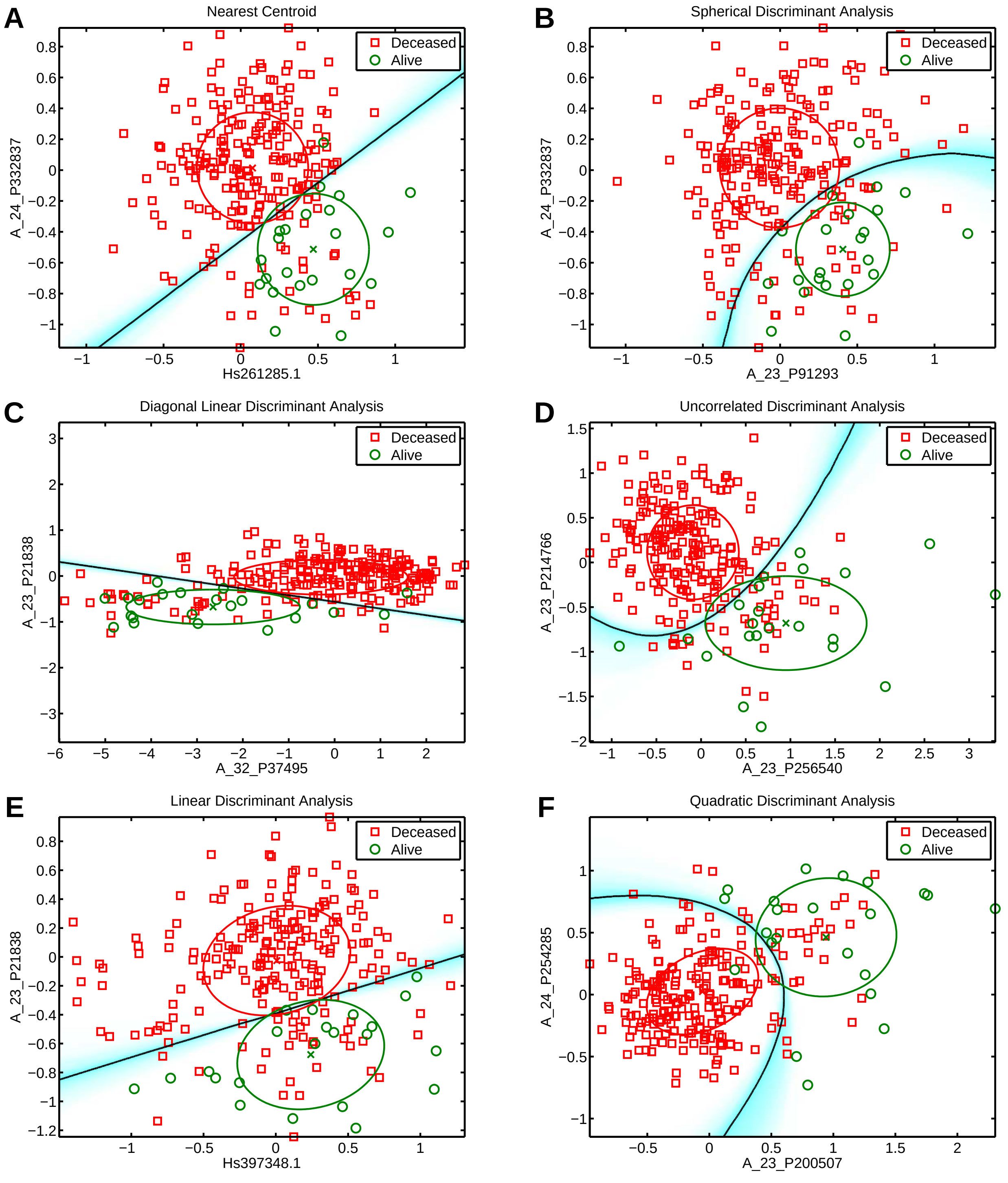

Figure S5 – Classifier discrimination plots for neuroblastoma, overall survival. Labels indicate whether or not the patient survived 900 days after diagnosis.

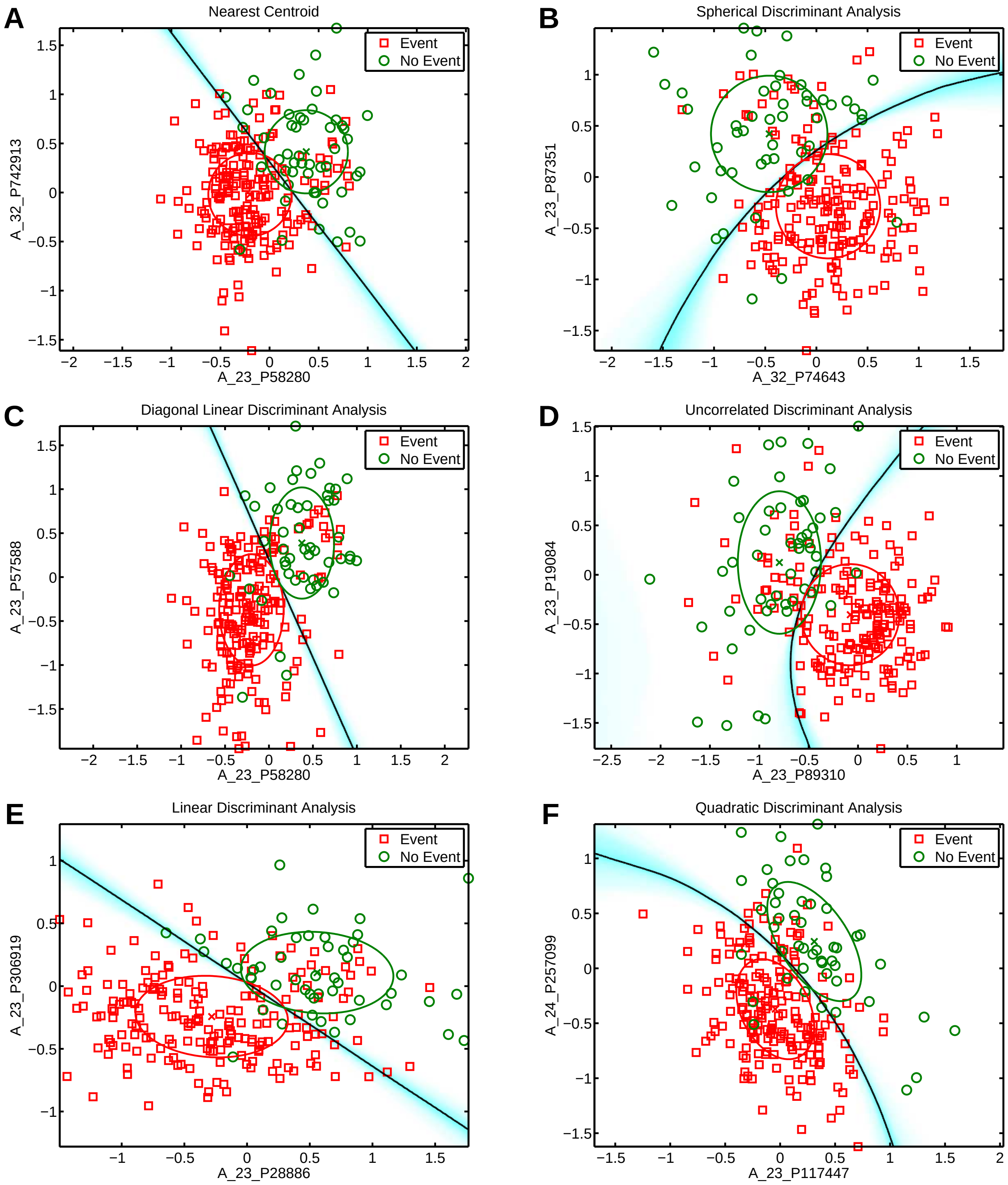

Figure S6 – Classifier discrimination plots for neuroblastoma, event-free survival. Labels indicate whether or not the patient survived without a medical event for 900 days after diagnosis.

Table S1 – **Estimated classifier performance for breast cancer, pathological complete response.**

Gene 1	Gene 2	NC	DLDA	LDA	SDA	UDA	QDA
217764_s_at	215127_s_at	0.812*	0.774	0.771	0.752	0.743	0.748
219233_s_at	205225_at	0.787	0.818*	0.770	0.781	0.799	0.775
216092_s_at	220040_x_at	0.785	0.787	0.796*	0.782	0.782	0.776
219438_at	218663_at	0.761	0.763	0.762	0.806*	0.777	0.771
203928_x_at	217297_s_at	0.739	0.744	0.759	0.793	0.810*	0.802
213228_at	212956_at	0.764	0.761	0.761	0.765	0.799	0.809*

*top performing classifier or any classifier that does not have a significantly lower mean using a paired t-test with $\alpha = 0.05$.

Table S2 – **Estimated classifier performance for breast cancer, estrogen receptor status.**

Gene 1	Gene 2	NC	DLDA	LDA	SDA	UDA	QDA
212956_at	208606_s_at	0.913*	0.887	0.889	0.903	0.890	0.889
201413_at	212956_at	0.893	0.916*	0.911*	0.898	0.909	0.899
212956_at	202854_at	0.892	0.892	0.905*	0.886	0.887	0.900*
209603_at	207588_at	0.890	0.895	0.893	0.915*	0.877	0.905
212956_at	214745_at	0.906	0.923	0.909	0.911	0.931*	0.922
39549_at	212956_at	0.925	0.911	0.927	0.925	0.910	0.933*

*top performing classifier or any classifier that does not have a significantly lower mean using a paired t-test with $\alpha = 0.05$.

Table S3 – **Estimated classifier performance for multiple myeloma, overall survival.**

Gene 1	Gene 2	NC	DLDA	LDA	SDA	UDA	QDA
22729	22429	0.688*	0.681	0.678	0.680	0.673	0.655
2319	1488	0.661	0.696*	0.689	0.666	0.678	0.665
46981	18436	0.667	0.712	0.718*	0.665	0.677	0.676
506	28077	0.656	0.672	0.673	0.716*	0.699	0.696
40394	8499	0.614	0.619	0.622	0.697	0.710*	0.687
9787	8499	0.620	0.620	0.624	0.648	0.675	0.691*

*top performing classifier or any classifier that does not have a significantly lower mean using a paired t-test with $\alpha = 0.05$.

Table S4 – **Estimated classifier performance for multiple myeloma, event-free survival.**

Gene 1	Gene 2	NC	DLDA	LDA	SDA	UDA	QDA
668	4289	0.683*	0.679*	0.678*	0.670	0.670	0.676
32757	13611	0.658	0.699*	0.695	0.654	0.687	0.682
1254	38113	0.638	0.688	0.692*	0.627	0.683	0.685
46271	23211	0.654	0.658	0.657	0.698*	0.691	0.694*
20930	18022	0.683	0.689	0.673	0.695	0.698*	0.667
27874	13611	0.682	0.666	0.674	0.700*	0.692	0.704*

*top performing classifier or any classifier that does not have a significantly lower mean using a paired t-test with $\alpha = 0.05$.

Table S5 – **Estimated classifier performance for neuroblastoma, overall survival.**

Gene 1	Gene 2	NC	DLDA	LDA	SDA	UDA	QDA
A_24_P332837	Hs261285.1	0.904*	0.894	0.894	0.901	0.893	0.895
A_23_P21838	A_32_P37495	0.781	0.895*	0.881	0.783	0.872	0.854
A_23_P21838	Hs397348.1	0.874	0.877	0.891*	0.859	0.846	0.873
A_24_P332837	A_23_P91293	0.865	0.865	0.865	8.890*	0.868	0.850
A_23_P214766	A_23_P256540	0.889	0.889	0.887	0.885	0.904*	0.865
A_24_P254285	A_23_P200507	0.828	0.850	0.829	0.885	0.866	0.896*

*top performing classifier or any classifier that does not have a significantly lower mean using a paired t-test with $\alpha = 0.05$.

Table S6 – **Estimated classifier performance for neuroblastoma, event-free survival.**

Gene 1	Gene 2	NC	DLDA	LDA	SDA	UDA	QDA
A_32_P742913	A_23_P58280	0.854*	0.844	0.843	0.852*	0.840	0.838
A_23_P57588	A_23_P58280	0.847	0.860*	0.845	0.846	0.856	0.841
A_23_P306919	A_23_P28886	0.794	0.859	0.865*	0.801	0.845	0.856
A_23_P87351	A_32_P74643	0.832	0.824	0.823	0.857*	0.840	0.850*
A_23_P19084	A_23_P89310	0.844	0.855	0.856	0.848	0.870*	0.861
A_24_P257099	A_23_P117447	0.818	0.847	0.863*	0.821	0.853	0.867*

*top performing classifier or any classifier that does not have a significantly lower mean using a paired t-test with $\alpha = 0.05$.
